# A rare case of a right common iliac venous aneurysm imitating a neoplastic tumour

**DOI:** 10.1186/s12872-018-0920-3

**Published:** 2018-09-25

**Authors:** Marcin Warot, Tomasz Synowiec, Błażej Kuffel, Patryk Szwarckopf, Maciej Micker, Paweł Chęciński

**Affiliations:** Department of General and Vascular Surgery and Angiology, Poznan University of Medical Sciences (PUMS), 34 Dojazd St, 60-631 Poznan, Poland

**Keywords:** Venous aneurysm, Neoplasm, Neoplastic, Computed tomography, Thromboembolic complication

## Abstract

**Background:**

Aneurysms of the deep lower limbs veins are extremely rare. Diagnosis of such conditions may be confusing and suggest the presence of a neoplastic lesion.

**Case presentation:**

We herein report a case of a 68-year-old woman who was admitted with a large vein tumour revealed by sonography and computed tomography. A direct phlebography revealed a large venous aneurysm of the right common iliac vein with an adhering thrombus and a large collateral circulation. Anticoagulant treatment and compression with an elastic stocking were initiated because the patient refused surgical treatment. A 2-year follow-up showed no aneurysm growth or thromboembolic complications.

**Conclusions:**

We show herein that conservative management can be effective and safe in cases of this rare condition.

## Background

Venous aneurysms are extremely rare anomalies within the veins. Superficial vein aneurysms may be misdiagnosed as inguinal hernias [1]. The authors of this manuscript conclude that the term “venous aneurysm” should be used only in relation to lesions located subfascially and all cases involving the dilatation of superficial veins should be called “varicose veins.”

Venous aneurysms are associated with deep vein thrombosis and pulmonary embolism. On radiologic examination (for example, ultrasound and computed tomography), deep vein aneurysms may be misdiagnosed as neoplastic tumours in the abdominal cavity.

Due to the rarity of the described pathology, a unified management protocol of such lesions has not yet been established. The treatment is mainly based on the clinicians’ experience.

## Case presentation

**Fig. 1:**
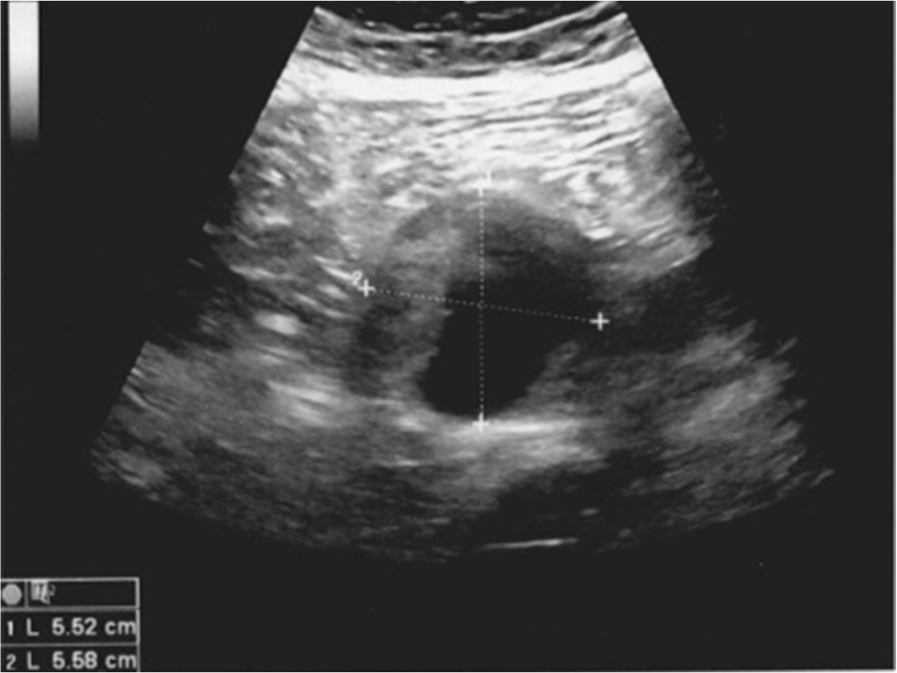
Ultrasound image of the tumour

A 68-year-old woman was admitted by her general practitioner (GP) with chronic pain situated in the epigastrium lasting for several years. No complaints of nausea, vomiting, body weight loss, or other symptoms were noted. The patient had no history of oedema of the lower limbs, varices, and trauma. Her only prior surgery was a Fothergill-Manchester operation in 2004 due to genital prolapse and urinary incontinence. The GP performed an abdominal ultrasound revealing a fluid-filled tumour (5.5 × 5.5 cm) situated between the common iliac arteries (Fig. 1).

**Fig. 2:**
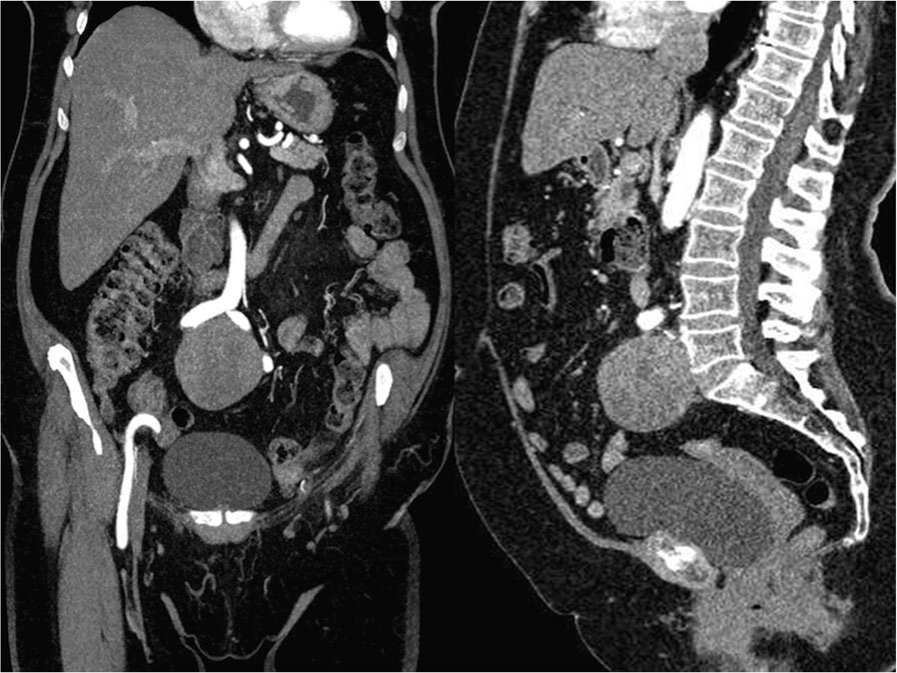
Tumour via CT scan

**Fig. 3:**
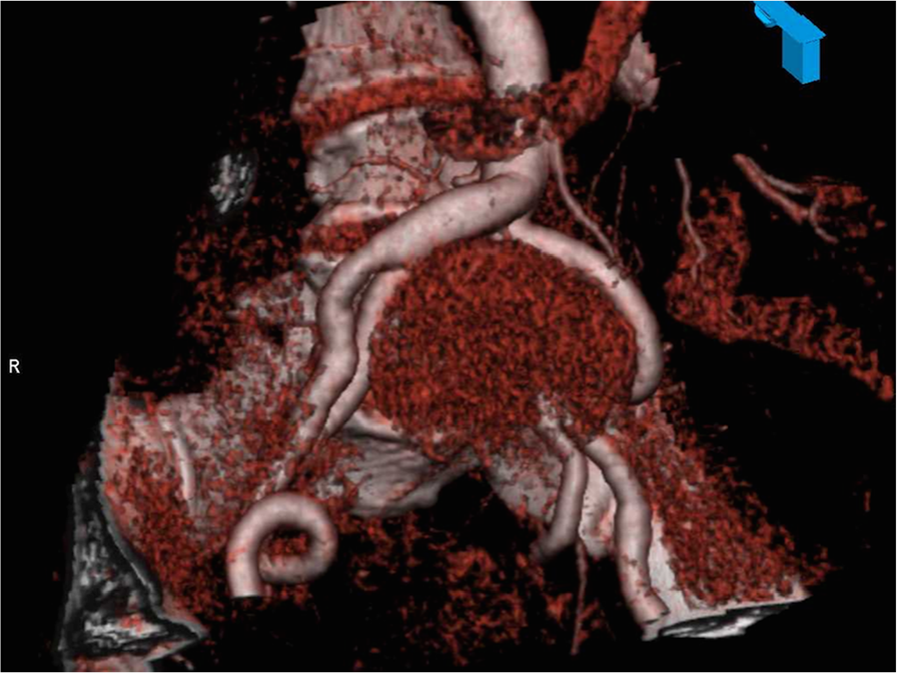
Tumour via 3D reconstruction

No pathology was noted during the complete gynaecological examination. Subsequently, computed tomography (CT) of the abdomen and pelvis was performed revealing an enormous mass in the presacral space. The CT image clearly suggested the presence of a neoplasm in the abdominal cavity (Figs. 2 and 3).

**Fig. 4:**
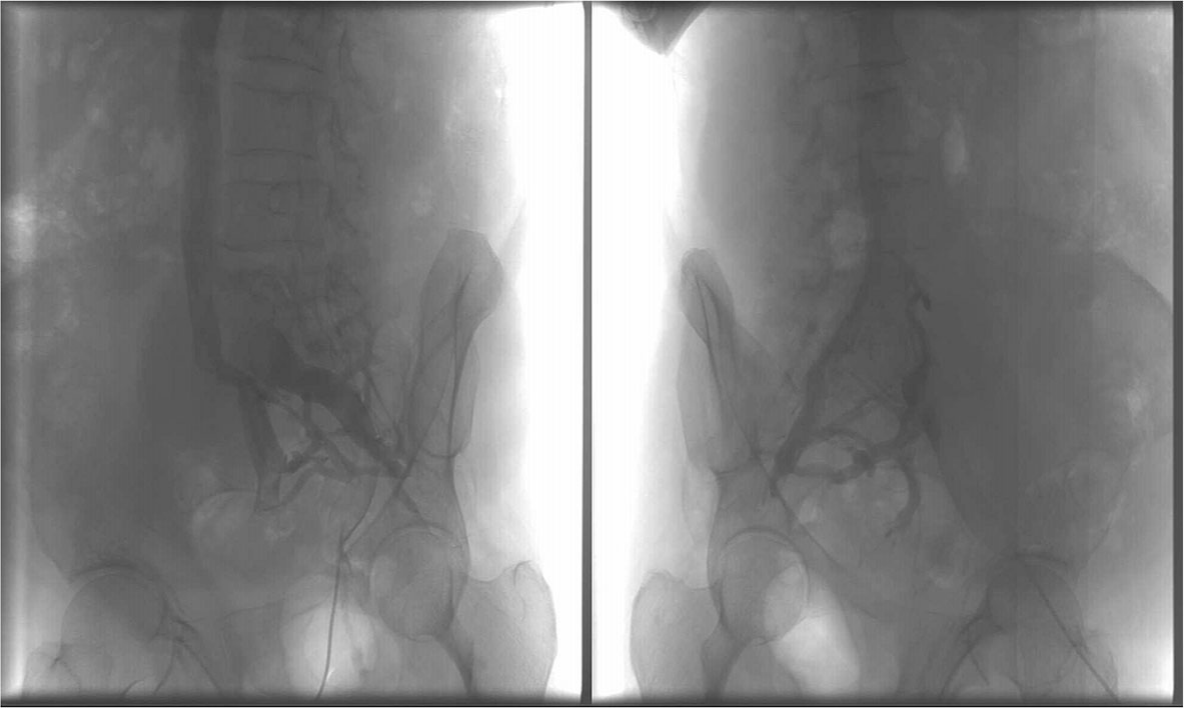
Venous aneurysm via direct phlebography

After the CT, the patient was admitted to the Department of General and Vascular Surgery and Angiology. An arterial and venous Doppler ultrasound was performed that indicated proper arterial flow and no deep vein thrombosis in the lower limbs. The Doppler ultrasound was followed by a direct phlebography that revealed a large venous aneurysm of the right common iliac vein with an adhering thrombus and a large collateral circulation (Fig. 4). All of the imaging studies excluded the presence of arteriovenous fistula. The D-dimer value was in the normal range. No other pathology was noted. The patient was offered surgical treatment but refused. Anticoagulation therapy with rivaroxaban was administered.

She received anticoagulant treatment for 2 years, and no thromboembolic complications or aneurysm growth has been noted thus far.

## Discussion and conclusions

The definition of venous aneurysm is in fact difficult to establish. The authors assume that the extension of the deep vein (for example, femoroiliac) should be called a “venous aneurysm.” On the contrary, dilatation of the superficial veins (for example, saphenous) is itself varicose. We agree with the definition presented in the Rutherford textbook that varicose veins are subcutaneous veins wider than 4 mm (clinical class C2) [2].

On the other hand, Hach defined a venous aneurysm as a limited venous dilatation with an increased risk of thrombosis. However, that might also apply to superficial veins [3]. It seems that the term “venous aneurysm” can make sense only for changes that occur in the subfascial region. Otherwise, it is difficult to delineate a clear border between the venous aneurysm and the varicose veins. The authors use the term “aneurysm” in relation to veins with a great simplification, knowing that this term is closely related to pathology in the arteries. According to research by Ramelet and Monti, the pathology described in our paper is called “widening” and not an “aneurysm” [4].

The natural history and indications for the treatment of venous aneurysms are ambiguous because of their rare occurrence. The face and neck region is the most frequent localisation; the least frequent are veins in the abdominal cavity (iliac veins and the inferior cava vein) and thorax (the superior cava vein or mediastinal venous aneurysms) [5]. In other research, peripheral veins (for example, popliteal) are the most common localisation of venous aneurysms [6, 7]. However, most authors agree that the intra-abdominal location is rare.

Due to the presence of the thrombus in the lumen of the venous aneurysm, it can potentially be a source of pulmonary embolism and cause life-threatening conditions. According to Abbott’s classification, venous aneurysms can be divided into primary and secondary [8]. Primary venous aneurysms are mainly caused by arteriovenous fistulas or anomalies that increase pressure in the venous system; secondary venous aneurysms are post-traumatic. Pregnancy and physical activity can affect the development of the disease, but these cases are extremely rare and poorly recognised [9]. In the case presented herein, there were no symptoms of portal hypertension or other pathology within the pulmonary or circulatory system that could have increased pressure in the vena cava. Considering there were no clinical signs of portal hypertension and normal liver function together with ultrasound and CT results did not indicate portal hypertension, additional imaging was not conducted. Echocardiography indicated no signs of right ventricular overload or pulmonary hypertension.

The most important factor in the presented pathology is thrombosis in the aneurysmal sac (such as in this case) that does not cause typical symptoms of limb oedema and pain. The other possible complication is aneurysm rupture [10]. The most common symptom of an aneurysm situated in the deep veins of the lower limbs is pain, unlike jugular or axillary vein aneurysms that are asymptomatic masses [11]. The possible occurrence of venous aneurysms should be considered in differential diagnoses of neoplastic tumours visualised by radiological examination.

The treatment is surgically based to reduce potentially life-threatening consequences such as the risk of rupture, thromboembolism, or other organ compression.

Surgery should be performed in a vascular division and is based on ligation of the aneurysm, tangential excision with lateral venorrhaphy, autologous vein patch, or complete resection with interposition grafting [12, 13].

Treating the described pathology is controversial due to the disease’s rare occurrence and the difficulty in reaching an acceptable consensus. Due to the risk of pulmonary embolism, most clinicians agree that surgery is the best form of therapy. The authors concur. In the described case, the patient did not agree to the procedure; hence, only conservative treatment was applied. We decided to present the results of conservative treatment because it is rarely utilised.

Because the patient refused operative treatment, we decided to administer permanent oral anticoagulant drugs and compression with an elastic stocking with regular follow-up visits, including yearly CT scans of the pelvis and lungs.

## Abbreviations

CT: Computed tomography
GP: General practitioner

## References

[1] Molgaard C, Yucel E, Waltman A (1992). Color Doppler flow imaging appearance of a popliteal venous aneurysm. J Vase Interv Radiol.

[2] Padberg F Jr. Classification and clinical and diagnostic evaluation of patients with chronic venous disorders. In: Rutherford R. Vascular Surgery Sixth Edition. Elsevier Saunders. 2005. p.2230–2240.

[3] Hach W. Różne choroby układu żylnego. In: Chirurgia żył. Galaktyka . 2007. p.374–376.

[4] Ramelet AA, Monti M. Wtórna niewydolność żylna niezwiązana z zakrzepicą i inne choroby żył. W: Flebologia Przewodnik. Via Medica, Gdańsk, 2003.p. 95–103.

[5] Calligaro KD, Ahmad S, Dandora R (1995). Venous aneurysms: surgical indications and review of the literature. Surgery.

[6] Audu CO, Boniakowski AE, Robinson S (2017). Internal iliac venous aneurysm associated with pelvic venous insufficiency. J Vasc Surg Venous Lymphat Disord.

[7] Perrin M (2006). Venous aneurysms. Phlebolymphology.

[8] Abbott OA, Leigh TF (1964). Aneurysmal dilatations of the superior vena caval system. Ann Surg.

[9] Parer JT, Lichtenberg ES, Callen PW (1984). Iliac venous aneurysm in a pregnant patient with a renal transplant. A case report. J Reprod Med.

[10] Forsberg JO, Bark T, Lindholmer C (1988). Nontraumatic rupture of the iliac vein. Eur J Vasc Surg.

[11] Gillespie DL, Villavicencio JL, Gallagher C (1997). Presentation and management of venous aneurysms. J Vasc Surg.

[12] Banno H, Yamanouchi D, Fujita H (2004). External iliac venous aneurysm in a pregnant woman: a case report. J Vasc Surg.

[13] Aldridge SC, Comerota AJ, Katz ML (1993). Popliteal venous aneurysm: report of two cases and review of the world literature. J Vasc Surg.

